# The Role of Adenine Nucleotide Translocase in the Assembly of Respiratory Supercomplexes in Cardiac Cells

**DOI:** 10.3390/cells8101247

**Published:** 2019-10-13

**Authors:** Rebecca M. Parodi-Rullán, Xavier Chapa-Dubocq, Roberto Guzmán-Hernández, Sehwan Jang, Carlos A. Torres-Ramos, Sylvette Ayala-Peña, Sabzali Javadov

**Affiliations:** 1 Department of Physiology, School of Medicine, University of Puerto Rico, San Juan, PR 00936-5067, USA; rebecca.parodi-rullan@temple.edu (R.M.P.-R.); xavier.chapa@upr.edu (X.C.-D.); roberto.guzman7@upr.edu (R.G.-H.); sehwan.jang@upr.edu (S.J.); carlos.torres27@upr.edu (C.A.T.-R.); 2 Department of Pharmacology and Toxicology, School of Medicine, University of Puerto Rico, San Juan, PR 00936-5067, USA; sylvette.ayala@upr.edu

**Keywords:** H9c2 cardiomyoblasts, mitochondria, adenine nucleotide translocase, respiratory supercomplexes, ETC complexes

## Abstract

Individual electron transport chain complexes have been shown to assemble into the supramolecular structures known as the respiratory chain supercomplexes (RCS). Several studies reported an associative link between RCS disintegration and human diseases, although the physiological role, structural integrity, and mechanisms of RCS formation remain unknown. Our previous studies suggested that the adenine nucleotide translocase (ANT), the most abundant protein of the inner mitochondrial membrane, can be involved in RCS assembly. In this study, we sought to elucidate whether *ANT* knockdown (KD) affects RCS formation in H9c2 cardiomyoblasts. Results showed that genetic silencing of ANT1, the main ANT isoform in cardiac cells, stimulated proliferation of H9c2 cardiomyoblasts with no effect on cell viability. *ANT1* KD reduced the ΔΨ_m_ but increased total cellular ATP levels and stimulated the production of total, but not mitochondrial, reactive oxygen species. Importantly, downregulation of *ANT1* had no significant effects on the enzymatic activity of individual ETC complexes I–IV; however, RCS disintegration was stimulated in *ANT1* KD cells as evidenced by reduced levels of respirasome, the main RCS. The effects of *ANT1* KD to induce RCS disassembly was not associated with acetylation of the exchanger. In conclusion, our study demonstrates that ANT is involved in RCS assembly.

## 1. Introduction

The adenine nucleotide translocase (ANT), one of the most abundant proteins of the inner mitochondrial membrane, exchanges the matrix ATP for ADP in the intermembrane space and thus, links mitochondrial ATP production with cellular energetics. Several studies have demonstrated a crucial role of ANT in the pathogenesis of cardiac diseases. Downregulation of ANT1, the main ANT isoform in the heart and skeletal muscle [1,2], has been found in patients with hypertrophic cardiomyopathy, and lactic acidosis [3]. Mice lacking *ANT1* developed cardiac hypertrophy and lactic acidosis [2], and a substantial decline in cardiac function compared to wildtype (WT) animals [4]. Heart- and muscle-specific *ANT1* knockout (KO) mice exhibit deficiency in mitochondrial bioenergetics associated with mitochondrial myopathy and hypertrophic cardiomyopathy [5]. Additionally, *ANT1* KO mice display an increase in reactive oxygen species (ROS) production and inhibition of oxidative phosphorylation (OXPHOS) in cardiac mitochondria [6]. Moreover, cardiac ischemia-reperfusion (IR) reduced *ANT1* expression whereas cardiac-specific overexpression of *ANT1* attenuated IR injury and reduced infarct size in rats [7]. In rat neonatal cardiomyocytes, overexpression of ANT1 protected against hypoxia-induced cell death, loss of mitochondrial membrane potential (ΔΨ_m_), and increased ROS production [7]. Therefore, understanding the role of ANT in the regulation of mitochondrial bioenergetics can provide a novel insight into mitochondrial-based cardiac therapies. 

ANT has been shown to interact with various subunits of the electron transport chain (ETC) complexes in HEK293 cells [8] and in yeast [9]. Several studies, the earliest one in 2000, demonstrated that ETC individual complexes can be assembled in large supramolecular structures known as respiratory chain supercomplexes (RCS) [10]. The main RCS is the respirasome, which is composed of complexes I, III, and IV in various stoichiometries. It has been proposed that the respirasome facilitates electron transfer, reduces electron leakage and mitochondrial ROS (mtROS) production, maintains structural organization of ETC complexes, and provides an efficient ATP production [11]. 

The assembly mechanisms and the structural identity of RCS remain to be elucidated. The role of ANT in RCS formation was recently proposed after it was observed that ANT interacts with RCS and that this interaction is conserved from yeast to higher eukaryotes [8], potentially implicating a crucial role of ANT in mitochondrial bioenergetics. However, these studies were mostly done in yeast and HEK293 cells; the RCS and ANT interactome has not been reported in mammalian tissues, particularly, in the heart. We have shown that pharmacological inhibition of ANT by atractyloside provoked RCS disintegration in cardiac mitochondria in vitro [12]. These studies suggest that ANT may have a structural interaction with RCS and/or play a regulatory role in RCS. Furthermore, post-translational modifications on ANT may affect its regulatory and structural capability in RCS assembly. Indeed, acetylation has been demonstrated to regulate the activity of ETC complexes [13,14] and thus, might affect the RCS stability. 

Here, we investigated the role of ANT1 in RCS assembly in H9c2 cardiomyoblasts. *ANT1* KD cells demonstrated increased total cellular ATP levels, with a reduction in ΔΨ_m_ and no changes in mitochondrial ATP production. However, *ANT1* KD did not affect the enzymatic activity of individual ETC complexes nor mitochondrial oxygen consumption. Deficiency in *ANT1* expression induced disassembly of RCS, particularly the respirasome, suggesting a potential role of ANT in RCS formation. Also, we found that ANT1 is not hyperacetylated in *SIRT3* KO mice although RCS levels in these animals were lower than in WT counterparts. 

## 2. Materials and Methods

### 2.1. Animals

Three-month-old male adult WT (129S1/SvImJ) and SIRT3^−/−^ (Sirt3^tm1.1Fwa^) mice (20–25 g) were purchased from Jackson Laboratory (Bar Harbor, ME, USA). All experiments were performed according to protocols approved by the UPR Medical Sciences Campus Animal Care and Use Committee and conformed to the National Research Council Guide for the Care and Use of Laboratory Animals published by the US National Institutes of Health (2011, eighth edition).

### 2.2. Cell Culture

H9c2 rat embryonic cardiomyoblast cells (American Type Culture Collection, Manassas, VA, USA) were cultured according to the manufacturer’s recommendations. The cells were cultured in high-glucose Dulbecco’s Modified Eagle’s Medium (DMEM, Sigma-Aldrich, St. Louis, MO, USA)-modified solution containing 4 mM L-glutamine, 4.5 g/L glucose, 1 mM sodium pyruvate, and 1.5 g/L sodium bicarbonate (Sigma-Aldrich, St. Louis, MO, USA) supplemented with 10% fetal bovine serum and 1% antibiotic solution (HyClone, GE Healthcare Bio-sciences, Pittsburgh, PA, USA). The cells were maintained in a humidified incubator containing 95% air and 5% CO_2_ at 37 °C.

### 2.3. siRNA Transfection

H9c2 cells were seeded for 40–60% confluency at 24 h. On the day of the experiment, cells were transfected using Lipofectamine RNAiMAX (Invitrogen, Thermo Fisher Scientific, Waltham, MA, USA) and FlexiTube small interfering RNA (siRNA, Qiagen, Germantown, MD, USA) according to the manufacturer’s recommendations. Briefly, H9c2 cells were seeded with Opti-MEM™ Reduced Serum Medium, GlutaMAX™ (Thermo Scientific, Thermo Fisher Scientific, Waltham, MA, USA) supplemented with 5% fetal bovine serum and 1% antibiotic solution to reach a 40–60% confluency in 24 h. On the next day, Lipofectamine RNAiMAX (Invitrogen, Thermo Fisher Scientific, Waltham, MA, USA) and FlexiTube siRNA mixtures were added. The following siRNA sequences (sense strand) were used: negative control (NC): UUC UCC GAA CGU GUC ACG, and ANT1: GAC GCA AAG CUU UCU UCA ATT. All experiments were conducted 48 h post-transfection.

Cell viability was determined by the trypan blue exclusion method using the TC20 Automated Cell Counter (Bio-Rad, Hercules, CA, USA).

### 2.4. Mitochondrial Oxygen Consumption Rate and ATP Production

Oxygen consumption rate and ATP production in H9c2 cells were determined using the Seahorse XFe24 analyzer (Agilent, Santa Clara, CA, USA). An equal number of cells were seeded and transfected at 24 h. Mitochondrial oxygen consumption rate and ATP production were determined 48 h post-transfection following manufacturer’s recommendations. Briefly, cell media was changed to Seahorse XF DMEM Medium, pH 7.4, and supplemented with (in mM): 10 glucose, 1 sodium pyruvate, and 2 L-glutamine. Mitochondrial functional parameters were determined after the addition of (in µM): 0.5 oligomycin, 4 carbonyl cyanide-4-(trifluoromethoxy)phenylhydrazone (FCCP), and 0.5 rotenone/antimycin A. Data were extracted using the Seahorse XFe24 report generator and normalized to total protein.

### 2.5. Isolation of Mitochondria 

H9c2 cells were trypsinized and pelleted at 200× *g* for 7 min. The pellet was resuspended in ice-cold sucrose buffer containing (in mM): 300 sucrose, 10 Tris-HCl, and 2 EGTA; pH 7.4. Cells were centrifuged at 2,500× *g* for 5 min at 4 °C, the pellet was resuspended in sucrose buffer and incubated on ice for 5 min. To disrupt the plasma membrane and expose mitochondria, cells were plunged using a 27G needle, until all cells were successfully lysed. The cell lysate was then centrifuged at 400× *g* for 5 min and the supernatant was collected. The mitochondria were concentrated by centrifugation at 10,000× *g* for 5 min and the final pellet was dissolved in sucrose buffer.

To isolate liver mitochondria, the liver tissue removed from WT and *SIRT3* KO mice was cut and homogenized using a Polytron homogenizer in 2 mL of ice-cold sucrose buffer containing: 300 mM sucrose, 20 mM Tris-HCl, and 2 mM EGTA. Homogenate was then centrifuged at 2,000× *g* for 3 min, to remove cell debris. The supernatant was then centrifuged at 10,000× *g* for 15 min to precipitate mitochondria. The final pellet was washed once with sucrose buffer by centrifugation at 10,000× *g* for 10 min. The mitochondria-enriched pellet was resuspended in 200 µL of sucrose buffer. 

### 2.6. Enzymatic Activity of ETC Complexes in Cultured Cells

Enzymatic activity of ETC complexes was determined as previously described [15], with minor modifications and normalized to mg of mitochondrial protein. All assays were performed at the SpectraMax Microplate Reader (Molecular Devices, San Jose, CA, USA) at 37 °C.

### 2.7. Total and Mitochondrial ROS Production in Cultured Cells

Total ROS and mtROS production were measured with 2′,7′-dichlorodihydrofluorescein diacetate (H_2_DCFDA) and MitoSOX Red, respectively [15]. Briefly, cells were incubated for 30 min with 10 µM H_2_DCFDA or 1 µM of MitoSOX and fluorescence intensity was monitored on the SpectraMax Microplate Reader (Molecular Devices, San Jose, CA, USA) at the excitation/emission of 599 nm/522 nm (for H_2_DCFDA) and 510 nm/580 nm (for MitoSOX). 

### 2.8. Mitochondrial Membrane Potential and Total ATP

To measure ΔΨ_m_ in cultured cells, H9c2 cells were incubated with ΔΨ_m_-sensitive dye JC-1 (5,5′,6,6′-tetraethyl-benzimidazolylcarbocyanine iodide; Molecular Probes, Thermo Fisher Scientific, Waltham, MA, USA). Briefly, cells were incubated for 30 min at 37 °C with JC-1 and fluorescence was measured using a SpectraMax Microplate Reader (Molecular Devices, San Jose, CA, USA). J-aggregates (red) and JC-1 dye monomers (green) were monitored at 530 and 590 nm emission (with excitation at 488 nm), respectively. Data are presented as the ratio red/green fluorescence.

ATP levels were measured using the ATP Bioluminescence Assay Kit CLS II (Roche, Indianapolis, IN, USA), according to the manufacturer’s recommendations. Luminescence data were normalized to total protein levels.

### 2.9. SDS-PAGE and Western Blotting

To analyze protein levels, equal amounts of protein were resolved by SDS-PAGE and transferred onto Amersham Hybond ECL nitrocellulose membranes (GE Healthcare Bio-sciences, Pittsburgh, PA, USA). The membranes were immunoblotted with antibodies against ANT1 (Abcam #110322, Cambridge, MA, USA), or ATP5a (Abcam #14748, Cambridge, MA, USA) followed by incubation with IRDye^®^ (LI-COR Biosciences, Lincoln, NE, USA) secondary antibodies. Bands were visualized using an ODYSSEY^®^ CLx (LI-COR Biosciences, Lincoln, NE, USA) infrared scanner. The resulting images were analyzed with Image Studio Lite Software version 5.2. 

### 2.10. Co-Immunoprecipitation

To analyze protein acetylation, immunoprecipitation experiments were performed following the recommended protocol of Dynabeads (Invitrogen-Life Technologies, Thermo Fisher Scientific, Waltham, MA, USA). Proteins containing acetylated lysine (Ac-K) residues were immunoprecipitated from mouse liver mitochondrial extracts using an antibody against acetylated lysine residues (Cell Signaling #9814, Danvers, MA, USA). The immunoprecipitates were separated by SDS-PAGE, blotted onto Amersham Hybond ECL nitrocellulose membranes (GE Healthcare Bio-Sciences, Pittsburgh, PA, USA) and the Western blots developed using antibody against ANT1 (Abcam #110322, Cambridge, MA, USA) and followed by secondary antibodies. Bands were visualized using the ODYSSEY^®^ CLx (LI-COR Biosciences, Lincoln, NE, USA) infrared scanner.

### 2.11. Analysis of RCS by Blue Native Polyacrylamide Gel Electrophoresis (BN-PAGE) 

The RCS in isolated mitochondria were analyzed by BN-PAGE [12,16]. Briefly, NC or ANT1 KD H9c2 mitochondrial protein or rat heart mitochondria treated for 45 min with vehicle (Veh, 0.01% DMSO), 500 nM rotenone (complex I inhibitor), 500 nM antimycin A (complex III inhibitor), or 1 µM FCCP were dissolved in solubilization buffer (50 mM NaCl, 50 mM imidazole-HCl, 2 mM 6-aminohexanoic acid, 1 mM EDTA) supplemented with digitonin, protease and phosphatase inhibitor cocktails (Sigma-Aldrich, St. Louis, MO, USA), and 25U benzonase. Native gels were stained with Coomassie brilliant blue G250 and visualized with the ODYSSEY^®^ CLx (LI-COR Biosciences, Lincoln, NE, USA) infrared scanner. The images were analyzed using Image Studio Lite Software. The respirasome levels were calculated as the pixel density of bands containing complex I, III, and IV and normalized to whole lane densities. 

### 2.12. Statistical Analysis

Data are presented as means ± SEM. Statistical significance was evaluated using Prism Graph Pad (San Diego, CA, USA) using an unpaired two-tailed Student’s *t*-test, Mann–Whitney test, or a one-way ANOVA. The BN-PAGE analysis was conducted with a repeated one-way ANOVA analysis. Differences were considered to be statistically significant when *P* < 0.05.

## 3. Results

### 3.1. ANT1 KD Increases Cellular Proliferation Without Affecting Cell Viability

**Figure 1:**
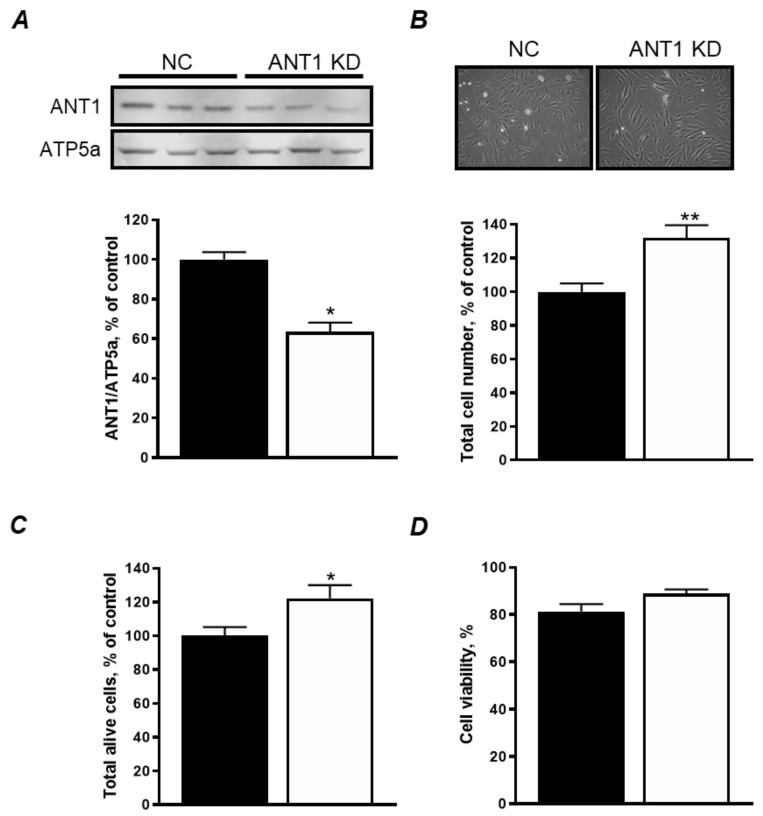
Cell viability is not affected by *ANT* KD in H9c2 cells. (**A**) Protein levels of ANT1 in negative control (NC) and *ANT1* KD cells. *Top panel:* representative immunoblots. *Bottom panel:* quantitative data of ANT1 protein expression normalized to ATP5a (a mitochondrial housekeeping protein); (**B**) Total number of cells 48 h after transfection. *Top panel:* representative images of cells. *Bottom panel:* quantitative data of cells; (**C**) total number of live cells 48 h after transfection; (**D**) cell viability 48 h after transfection calculated as (alive cells/total cells) × 100. * *P* < 0.05 and ** *P* < 0.001 vs. NC. Data represent 4–7 independent experiments.

Transfection with *ANT1* siRNA significantly reduced ANT1 expression by 37% (*P* < 0.001) 48 h after transfection (Figure 1A). Interestingly, we found that *ANT1* KD increases cell number by 32% (*P* < 0.001, Figure 1B) and the number of alive cells by 22% (*P* < 0.05, Figure 1C) without affecting cell viability (Figure 1D). Altogether, these results suggest that *ANT1* KD does not affect cell viability, but it increases cellular proliferation, possibly as an adaptive response to ANT1 downregulation.

### 3.2. ANT1 KD Increased Total ATP and ROS Levels with no Effect on the ETC Activity and mtROS Production 

**Figure 2:**
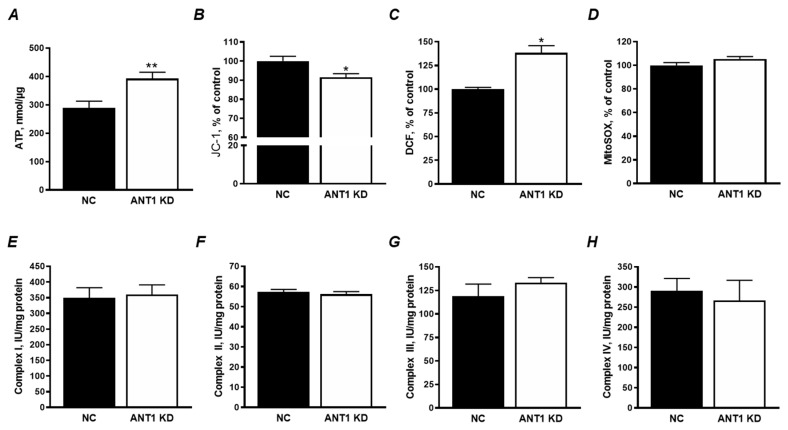
*ANT1* KD disturbs mitochondrial membrane potential (ΔΨm) without affecting enzymatic activity of ETC complexes. (**A**) Total cellular ATP levels normalized to the total amount of protein. (**B**) Mitochondrial membrane potential determined with JC-1 after transfection and calculated as the ratio of J-aggregates to JC-1 monomers. (**C**) Total cellular ROS assessed with H_2_DCFDA; (**D**) MtROS assessed using MitoSOX red. Data on the fluorescence activity in the cells (**B**–**D**) are presented as percent change of negative control (NC). (**E**–**H**) The enzymatic activity of complexes I, II, III and IV in mitochondria isolated from NC and *ANT1* KD cells. Data were normalized to mitochondrial protein levels. * *P* < 0.05, and ** *P* < 0.01 vs. control (NC). Data represent 3 independent experiments.

Total ATP levels were elevated by 36% (*P* < 0.01) in *ANT1* KD cells (Figure 2A). Total ATP levels were normalized to µg of total cellular protein to account for the observed increase in cell number (Figure 1B). However, it should be noted that this method does not distinguish glycolytic from mitochondrial ATP. Although these cells appear to have higher levels of total ATP, a decrease in ΔΨ_m_ could hint towards a higher glycolytic ATP production. Results showed that *ANT1* KD cells had significantly lower ΔΨ_m_ compared to NC cells (Figure 2B), suggesting that the elevated ATP levels might result from increased glycolysis but not OXPHOS. 

Next, we examined total ROS production using H_2_DCFDA fluorescent dye. Results demonstrate that *ANT1* KD cells have a 38% increase (*P* < 0.001) in total ROS generation when compared to NC (Figure 2C). Analysis of mtROS production by MitoSOX shows that *ANT1* KD has no effect on mtROS (Figure 2D). The lack of a difference in mtROS levels can be explained with no significant electron leakage due to low ETC flow in *ANT1* KD cells. In favor of this, analysis of the enzymatic activity of the ETC complexes I, II, III, and IV demonstrated no difference between *ANT1* KD and control cells (Figure 2E–H). Our data are consistent with previous studies where the activity of complexes I, III, and IV were unaffected by ANT expression in HEK293 cells [8]. Altogether, these data demonstrate that ANT1 deficiency has no effect on the enzymatic activity of individual ETC complexes and mtROS production.

### 3.3. Mitochondrial Oxygen Consumption Rate and OXPHOS is not Affected by ANT1 Downregulation

**Figure 3:**
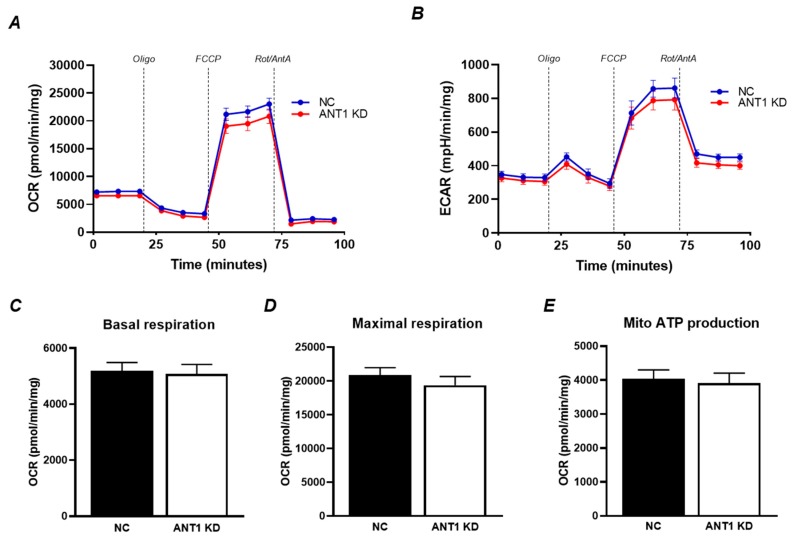
Mitochondrial oxygen consumption and ATP production is not affected by ANT1 downregulation. (**A**) oxygen consumption rate (OCR); (**B**) extracellular acidification rate (ECAR); (**C**) basal respiration; (**D**) maximal respiration; (**E**) mitochondrial ATP production. All parameters were determined using the Seahorse XFe24 analyzer (Agilent) after the addition of (in µM): 0.5 oligomycin (Oligo), 4 FCCP, and 0.5 rotenone/antimycin A (Rot/AntA). The data was extracted using the Seahorse XFe24 report generator and normalized to total protein levels. Data represent 3 independent experiments.

We measured mitochondrial oxygen consumption rate (OCR) and extracellular acidification rate (ECAR) in H9c2 cells treated with scrambled and *ANT1* siRNA using the Seahorse XFe24 analyzer. Results demonstrate that *ANT1* silencing does not affect the OCR and ECAR in these cells (Figure 3A,B). Likewise, basal and FCCP-induced maximal respiration rates were found unchanged in *ANT1* KD cells (Figure 3C,D). As expected, mitochondrial ATP production was not affected by ANT1 downregulation (Figure 3E). Altogether, these results demonstrate that *ANT1* silencing does not affect OXPHOS in H9c2 cells.

### 3.4. ANT1 KD in H9c2 Cells Induce RCS Dissociation: the Role of Acetylation

**Figure 4:**
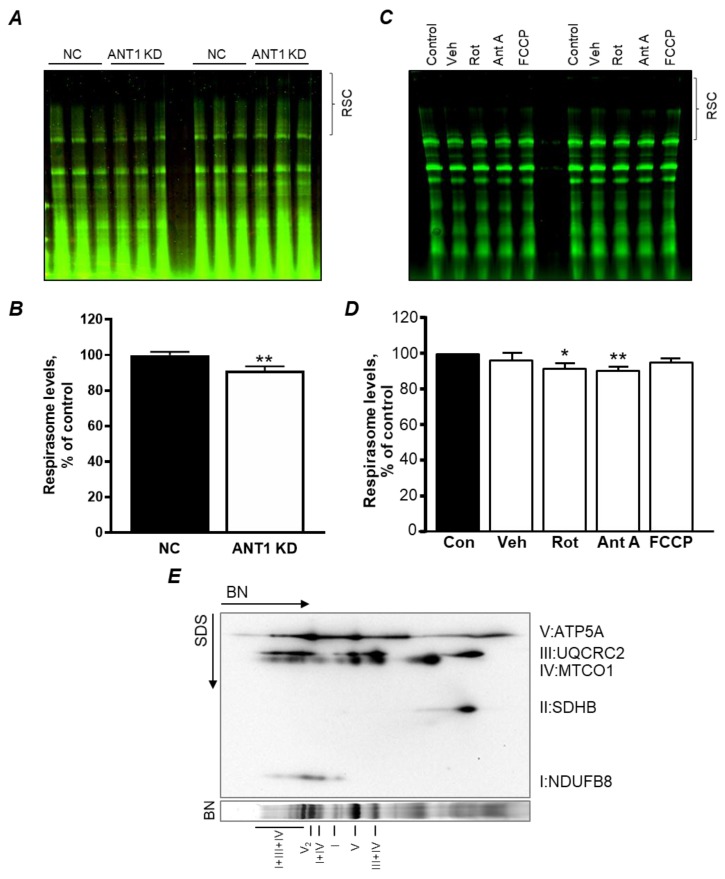
*ANT1* KD stimulates mitochondrial respirasome disintegration in H9c2 cells. (**A**) representative blue native (BN) gel of mitochondria isolated from *ANT1* KD cells and subjected to BN-PAGE; (**B**) quantitative data of respirasome levels; (**C**) representative BN-PAGE gel of mitochondria isolated from rat heart and treated with vehicle (Veh, 0.01% DMSO), 500 nM rotenone (Rot), 500 nM antimycin A (Ant A), or 1 µM FCCP; (**D**) quantitative data of respirasome levels in the groups shown in C; (**E**) representative two-dimensional BN-PAGE of ETC complexes in mitochondria isolated from the rat heart. RCS were analyzed in mitochondria where membrane proteins were solubilized using digitonin and separated by BN-PAGE. ETC complexes were visualized using specific antibodies against the subunits for complexes I (NDUFB8), II (SDHB), III (UQCRC2), IV (MTCO1), and V (ATP5A). Respirasome is shown as I+III+IV. Data in B and D were normalized to mitochondrial protein levels and presented as percent change of negative control (NC) or control (Con). * *P* < 0.05 and ** *P* < 0.01 vs. NC or Con. Data represent 3–4 independent experiments.

Our recent studies [12] demonstrated that pharmacological inhibition of ANT by atractyloside induces RCS dissociation in isolated cardiac mitochondria. Analysis of RCS in scrambled (NC) or *ANT1* siRNA-treated H9c2 cells demonstrated that ANT1 deficiency induces disassembly of respirasome by 9% (*P* < 0.01) compared to control cells (Figure 4A,B), suggesting that ANT is involved in RCS integrity and stabilization. In order to validate that the decrease in RCS was due to ANT1 downregulation but not ΔΨ_m_ loss (Figure 2B), mitochondria isolated from rat hearts were treated with 1 µM FCCP, an uncoupler, for 45 min [12], prior to RCS analysis. Results demonstrate that loss of ΔΨ_m_ does not affect RCS integrity (*P* < 0.1473, Figure 4C,D). Taken together, these results suggest that the loss of RCS in *ANT1* KD cells is not due to the loss of ΔΨ_m_ and may result from the downregulation of ANT1.

**Figure 5:**
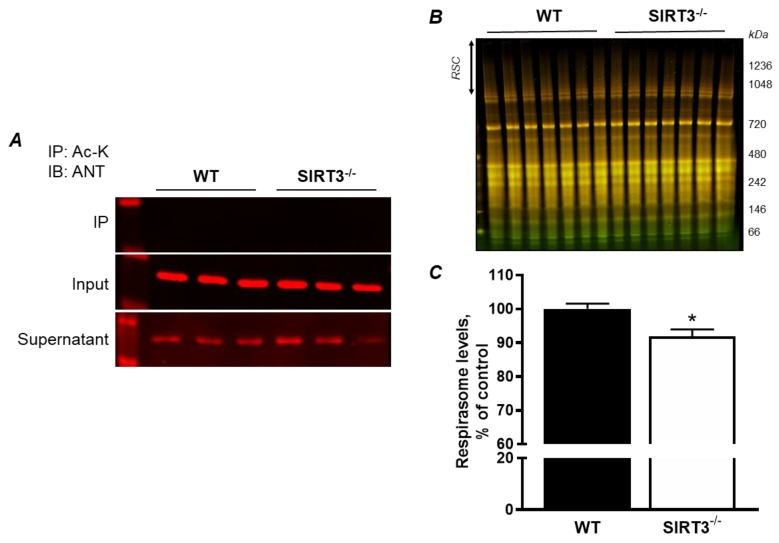
ANT1 is not acetylated but acetylation of mitochondrial proteins stimulates RCS disassembly in *SIRT3* KO mitochondria. (**A**) Immunoprecipitation (IP) of liver mitochondrial proteins with acetylated lysine (Ac-K) antibodies followed by immunoblotting (IB) against ANT1. *Input:* the sample before IP; *Supernatant:* sample that did not bind to Ac-K antibodies; (**B**) BN-PAGE gel of liver mitochondria isolated from WT and *SIRT3* KO^−^ mice. (**C**) Quantitative data of respirasome levels. The data were normalized to total protein levels and presented as percent change from the WT group. * *P* < 0.01 vs. WT; n = 6–7 animals per group.

In the following set of experiments, we examined whether ANT1 acetylation is involved in RCS formation. First, we analyzed liver mitochondria isolated from WT and *SIRT3* KO mice to determine the acetylation of total mitochondrial proteins. SIRT3 is the main mitochondrial isoform of sirtuins. Hyperacetylation of mitochondrial proteins due to SIRT3 deficiency has been shown to associate with cardiovascular, neurodegenerative diseases, diabetes, and aging [17,18,19]. We have previously demonstrated that SIRT3 ablation enhances lysine acetylation (Ac-K) of mitochondrial proteins [20]. However, immunoprecipitation analysis revealed no changes in ANT1 acetylation in the mitochondria of *SIRT3* KO mice (Figure 5A). Interestingly, the mitochondria of *SIRT3* KO mice demonstrated lower RCS levels compared to the WT group (Figure 5B,C). These results suggest that acetylation of mitochondrial proteins, but not ANT1, can stimulate RCS disassembly in mitochondria. 

## 4. Discussion

The ANT has an important role in maintaining mitochondrial bioenergetics [21] and recently, it has been proposed to play a role in RCS formation [8]. Therefore, in this study, we sought to clarify whether genetic downregulation of ANT1, the main isoform of ANT found in the heart and skeletal muscle cells [1], affects RCS assembly in H9c2 cardioblasts. Our results demonstrate that ANT1 downregulation by 37% does not affect cell viability with no remarkable changes in mitochondria bioenergetics. Furthermore, the activity of all ETC complexes and the mitochondrial OCR was not dependent on ANT1; however, ANT1 appears to be important in the assembly (structural integrity) of the RCS, particularly the respirasome. Additionally, we demonstrate that hyperacetylation of mitochondrial proteins due to SIRT3 ablation stimulates RCS disassembly. The novel role of acetylation on RCS stability may provide additional information as to the mechanism of how acetylation of mitochondrial proteins is involved in the pathogenesis of cardiovascular diseases such as hypertrophy [22,23,24], IR [20,25,26] and heart failure [27,28]. The current study was performed in H9c2 cardiomyoblasts, but not in primary cardiomyocytes because the latter are quite sensitive to genetic manipulations. It should be noted that H9c2 cardiomyoblasts are more energetically similar (at least, in comparison with atrial HL-1 cells) to primary cardiomyocytes and can be successfully used to simulate an in vitro model of cardiac diseases [29].

Apparently, the role of ANT in the regulation of RCS assembly is not associated with its acetylation as *SIRT3* KO did not increase ANT acetylation in liver mitochondria. Interestingly, we are the first to demonstrate that acetylation per se affects RCS assembly, which could contribute to the mitochondrial dysfunction observed in *SIRT3* KO hearts [20,25]. Disruption of the ANT has been linked to various cardiac diseases. In a mouse model of IR, ANT1 expression was significantly reduced, and cardiac-specific ANT1 overexpression prevented the detrimental effects associated with IR injury [7]. *ANT1* KO mice develop cardiac hypertrophy and lactic acidosis [2], similar to that observed in patients. Therefore, ANT1 has an important role in maintaining cardiac function and potentially mediating the detrimental effects associated with heart IR injury [30]. 

In our studies, *ANT1* KD increased cell number without affecting cell viability (Figure 1B–D). Previous studies have observed an increase in mitochondrial number, size [2,31], and upregulation of mitochondrial genes, including OXPHOS components [31] in *ANT1* KD hearts and skeletal muscle. It is tempting to speculate that *ANT1* KD cells display an increase in cellular proliferation as a reflection of upregulated mitochondrial genes and an increase in mitochondrial number and size as an adaptive response. The lack of any effects of ANT deficiency on cell viability might be explained, at least in part, by (i) insufficient (37%) downregulation of ANT1 to induce mitochondrial dysfunction, or (ii) upregulation of other ANT isoforms, such as ANT2, as a compensatory mechanism, and their functional redundancy. Indeed, ANT2 has been shown to have opposite properties to ANT1 as it has been found capable of importing cytosolic ATP into the mitochondrial matrix [32], possibly maintaining normal mitochondrial function, although these findings are somewhat controversial [33]. In addition, ANT2 is regarded as a proliferative marker and correlated to loss of cell cycle control, which could partially explain why *ANT1* KD cells have an increase in cell number [32]. 

Interestingly, *ANT1* KD increased the number of total cells by 32% and alive cells by 22% (Figure 1) associated with a 36% increase of ATP levels (Figure 2B). The increase of ATP levels in *ANT1* KD cells might be due to the increase in cell number; however, this suggestion was excluded after normalization of ATP to total cellular protein (Figure 2B). Since aerobic (non-glycolytic) ATP production is coupled to ΔΨ_m_, we sought to examine the possibility of having disturbances in mitochondrial ATP production that could hint towards a glycolytic compensation. Previous studies have reported an increase in anaerobic metabolism and lactic acidosis [2,3] in ANT1 deficiency. Our results demonstrated that *ANT1* KD cells display a decrease in ΔΨ_m_ (Figure 2B), which could be due to an impaired ETC activity and OXPHOS. However, neither we (Figure 2E–H) nor other groups using HEK293 cells [8] reported differences in the activity of individual ETC complexes due to ANT1 downregulation or ablation. In addition, we were unable to detect differences in basal and maximal mitochondrial respiration (Figure 3C,D) and ATP production (Figure 3E). Interestingly, although beyond the scope of our experiments, *ANT1* KD cells displayed a significant increase in cellular ROS levels (Figure 2C) and non-mitochondrial oxygen consumption (*data not shown)*. The production of ROS can occur outside the mitochondria, such as in the cytosol (xanthine oxidase, nitric oxide synthase), peroxisomes, and plasma membrane (NADPH oxidases) [34], possibly suggesting a cross-talk between ANT1-deficient mitochondria and other cellular compartments.

The physiological significance of the RCS is still under debate [35]. The mitochondrial RCS have been suggested to increase the effectiveness of electron transport through the ETC complexes, optimize ATP production, and reduce mtROS production by reducing electron leakage [11]. Disassembly of the RCSs, particularly the respirasome, was observed in cardiovascular diseases such as IR [16] and heart failure [36]. However, the mechanisms underlying the assembly of the RCSs, as well as their physiological role in the heart, are not fully understood. Our previous studies demonstrated that high Ca^2+^ and pharmacological/genetic inhibition of complex I (Figure 4C,D) stimulate disruption of the RCS in H9c2 cells and isolated mitochondria [12,37]. These studies suggested crosstalk between RCS assembly and permeability transition pore (PTP) opening as Ca^2+^ is the strong inducer of pore opening and complex I is the PTP regulator. This point is further supported by the current study that demonstrates that genetic downregulation of ANT, a PTP regulator, induces disorganization of the RCS. However, the cause–effect relationship between RCS and PTP seems to be more complex. Despite RCS disassembly, inhibition of complex I by rotenone prevented Ca^2+^-induced PTP opening in cardiac mitochondria [12], and *ANT1* KD did not increase mtROS, a PTP inducer in H9c2 cells (Figure 2D). Finally, we demonstrate that acetylation of mitochondrial proteins due to SIRT3 deficiency induces RCS disassembly in an ANT-independent manner because there was no difference in ANT acetylation between WT and SIRT3^−/−^ mitochondria (Figure 5). Disruption of the RCS could be a result of direct mechanisms involving disruption of protein–protein interactions due to changes in lysine residue charges, or indirect mechanisms through inactivation of RCS regulatory proteins (e.g., RCS assembly factors) due to their hyperacetylation.

In conclusion, this study suggests that ANT is involved in RCS assembly, although RCS may not be solely dependent on ANT. ANT may physically interact with ETC complexes I, III, and IV [8] and thus, be involved in the respirasome structure or play a regulatory role in the formation/maintenance of the RCS assembly. Further studies are required to elucidate the role of ANT in the structural integrity and regulation of RCS and other mitochondrial supercomplexes (e.g., ATP synthasome) in cardiac cells.

## 5. Limitations of the Study

We elucidated the contribution of only *ANT1* downregulation to mitochondrial bioenergetics and RCS assembly. ANT family proteins contain four isoforms (ANT1-4) that play a differential role and perform distinctly opposite functions in cell life and death. We were not able to verify protein expression of other ANT isoforms in *ANT1* KD H9c2 cells due to lack of reliable ANT2, ANT3, and ANT4 antibodies. Functional redundancy of other ANT isoforms could compensate for the effects induced by *ANT1* deficiency.
